# Role of Elevated Serum TGF-β1 and the Common Promoter *TGFB1*-509C/T Polymorphism in the Development and Progression of Primary Glial Tumors and Brain Metastases

**DOI:** 10.3390/medicina60010146

**Published:** 2024-01-13

**Authors:** Elina Aleksandrova, Ivan Mindov, Bozhidar Petrov, Ivelina Dimitrova, Nikolay Petrov, Julian Ananiev, Tatyana Vlaykova, Stefan Valkanov

**Affiliations:** 1 Department of Medical Chemistry and Biochemistry, Medical Faculty, Trakia University, 6000 Stara Zagora, Bulgaria; nikolay.petrov.mf.21@trakia-uni.bg (N.P.);; 2 Department of Surgery, Neurosurgery, Urology and Anaesthesiology, Medical Faculty, Trakia University, 6000 Stara Zagora, Bulgariabozhidar.petrov@trakia-uni.bg (B.P.); ivelina.dimitrova@trakia-uni.bg (I.D.); stefan.valkanov@trakia-uni.bg (S.V.); 3 Department of General and Clinical Pathology, Forensic Medicine, Deontology and Dermatovenerology, Medical Faculty, Trakia University, 6000 Stara Zagora, Bulgaria

**Keywords:** glial tumors, brain metastases, TGF-β1, polymorphism, immunohistochemistry

## Abstract

*Background and Objectives*: The role of transforming growth factor-beta1 (TGF-β1) has been widely studied in the context of carcinogenesis. It has been involved in the pathogenesis of primary brain tumors or brain metastases due to its pleiotropic effects on immune regulation and tissue homeostasis. In line with recent findings, the aim of the current study was to examine the role of circulating TGF-β1 and the -509C/T functional polymorphism (rs1800469) in the *TGFB1* gene promoter in the susceptibility and progression of primary brain tumors and brain metastases among patients from the Bulgarian population. *Materials and Methods*: Cases with a confirmed diagnosis were genotyped by the polymerase chain reaction-restriction fragment length polymorphism assay (PCR-RFLP). Serum TGF-β1 levels were determined by ELISA. Immunohistochemical evaluation of the expression of TGF-β1 and the TGF-β1 receptor-type II was conducted. *Results*: We observed that TGF-β1 serum levels correlate with the genotype and are sex-related. TGF-β1 serum levels were significantly elevated in patients compared to controls. Additionally, the T/T-genotype determined higher circulating levels of the cytokine. The same genotype determined the shorter median survival after surgery for the patients. The immunohistochemical analysis revealed a statistical tendency: cases expressing TGF-β1 in the cytoplasm had elevated levels of the cytokine in the serum compared to the negative cases. *Conclusions*: Overall, our results indicate a negative effect of the T-allele on the predisposition and prognosis of brain malignancies, and the genetically determined higher TGF-β1 serum levels might contribute to the worse prognosis and metastatic capacity of brain malignancies.

## 1. Introduction

Brain malignancies, including primary glial tumors and metastases of different origins, remain a challenge in neuropathology, leading to significant cancer-related mortality worldwide. A number of possible risk factors have been studied, but for a few, a consensus has been reached. Novel biomarker molecules and factors underlying carcinogenesis are being searched to elucidate the molecular pathogenesis of these tumors.

The transforming growth factor-beta 1 (TGF-β1) is a multifunctional molecule involved in various cellular processes such as proliferation, differentiation, extracellular matrix production, and tissue homeostasis [1]. Immune cells are one of the sources of TGF-β1, which is considered a cytokine with pleiotropic activity that is important for maintaining normal immune homeostasis [2]. It exerts highly pronounced anti-inflammatory and immunosuppressive functions [3], the latter being realized by controlling the activation, proliferation, differentiation, and survival of all effector cells of the immune system [4]. However, not all of the effects of TGF-β1 have anti-inflammatory and immunosuppressive properties. TGF-β1 can induce rapid accumulation of macrophages and granulocytes at the site of inflammation, possessing chemoattractive properties even at very low concentrations [5], and is able in combination with IL-6 to induce differentiation of Th17 cells [6]. The latter produce large amounts of IL-17 and maintain acute inflammation. This dual role of TGF-β1 in the course of the immune response should be considered when analyzing its involvement in different pathological conditions, including tumor pathogenesis.

TGF-β1 belongs to a large family of proteins with three isoforms expressed in mammalian cells, namely TGF-β1, TGF-β2, and TGF-β3. The TGF-β1 isoform is one of the most extensively studied. In the human genome, it is encoded by a gene located on the long arm of chromosome 19 (19q13.1-3). The gene encompasses a region of 23,020 bases (bp; base pairs) and contains 7 exons and 6 introns. The *TGFB1* promoter region is highly polymorphic. Several single nucleotide polymorphisms (SNPs) have been identified before the transcription start site in the gene [7], of which one of the best studied is the -509C/T polymorphism (rs1800469). This SNP affects the transcriptional activity of *TGFB1* and, consequently, the circulating levels of the protein itself. The molecular mechanism involves the transcription factor AP1 (activator protein-1), which inhibits the expression of a number of genes. In this case, the *TGFB1*-509C allele selectively attracts AP1 instead of HIF-1 to the promoter, which inhibits gene expression [8].

As a prominent immune regulatory cytokine, TGF-β1 is widely studied in relation to its role in the pathogenesis and progression of various types of cancer, including colorectal cancer [9,10,11], hepatocellular carcinoma [12], brain cancer [13], etc. Both pro- and antitumor effects of TGF-β1 have been reported, supporting the dualistic role of this cytokine in carcinogenesis. In the normal epithelium, TGF-β1 acts as a growth inhibitor, but it has been demonstrated that transformed tumor cells overexpress TGF-β1 and remain irresponsive to its growth-inhibitory effects [14]. It is also worth mentioning the role of the cytokine in the epithelial-mesenchymal transition (EMT) process. The EMT process, driven and regulated by TGF-β1, results in a significant phenotypic change in epithelial morphology [15], including enhanced migratory properties, invasiveness, and resistance to apoptosis [16].

TGF-β1 regulates cellular processes by binding to three high-affinity cell surface receptors, also known as receptors I, II, and III. The TGFβ-RI and TGFβ-RII receptors contain a serine-threonine tyrosine kinase in their intracellular domains that initiates intracellular signaling by phosphorylating several transcription factors known as Smads [17]. The signal transduction mechanism of TGF-β1 is expressed by direct binding of the protein to the type III receptor, which is then presented to the type II receptor, or it may directly bind to the type II receptor. Once activated by TGF-β1, the novel receptor II binds to and transphosphorylates the type I receptor, thereby stimulating its protein kinase activity. Thus, the activated type I receptor phosphorylates Smad2 and Smad3 proteins, which subsequently bind to Smad4. The result is a Smad complex that translocates to the nucleus, where it interacts specifically with various transcription factors regulating the transcription of multiple genes [18].

Recently, the role of TGF-β1 in the molecular pathogenesis of brain tumors has been extensively studied. Although generally considered a neuroprotector [19], TGF-β1 might be involved in the promotion and migration of glioma cells [20] and in selectively inducing self-renewal of the glioma-initiating cells via the SMAD-dependent activation of leukemia inhibitory factor (LIF) and the sequential activation of the LIF-Janus kinase-STAT pathway [21]. Additionally, it has been reported that TGF-β1 participates in mechanisms that create a tumor microenvironment permissive to metastatic dissemination [22], mostly via cancer-associated fibroblasts (CAFs), which provide cancer cells with proliferative, migratory, survival, and invasive capacities [23].

In line with the above findings, we aimed to explore the role of the *TGFB1*-509C/T SNP in the genetic predisposition to brain tumors and its association with circulating levels and tissue expression of TGF-β1 and the clinical parameters of patients from the Bulgarian population who were operated on high-grade primary glial tumors or brain metastases.

## 2. Materials and Methods

### 2.1. Study Subjects

A group of 61 patients with high-grade glial tumors or brain metastases were included in the present study. The diagnosis was confirmed histologically at the University Hospital “Prof. St. Kirkovich”, Stara Zagora, Bulgaria. The group was composed of 40 (65.6%) male and 21 (34.4%) female individuals with a mean age of 63.9 (±9.05) and 58.5 (±15.35), respectively, without significant age differences (*p* = 0.151, *t*-test). The mean age of the total group was 62.1 years (±11.76). According to the diagnosis, the patient group consisted of 31 high-grade glial tumors (2 cases were classified as Grade III and 29 as Grade IV) and 30 cases of brain metastases. The clinicopathological characteristics of the studied patients are listed in Table 1.

A group of 176 cancer-free controls from the same region as the patients were enrolled in the present study. The group consisted of 114 (65.5%) male and 60 (34.5%) female individuals. The mean age of the group was calculated as 63.15 (±11.32). There were no significant age differences between the male controls and females, with mean ages of 62.73 (±11.07) and 64.02 (±11.89), respectively (*p* = 0.486, *t*-test).

The tissue samples were collected in accordance with the ethical standards laid down in the 1964 Declaration of Helsinki. Our study was approved by the Local Ethic Committee of the Medical Faculty, Trakia University, Stara Zagora, Bulgaria (permission no. 9/15 May 2019).

### 2.2. Blood Samples and DNA Extraction

Blood samples were collected for serum separation and DNA isolation by appropriate blood collectors from all included subjects. Serum samples were frozen in small aliquots at −80 °C until analysis. Genomic DNA was extracted from 2 mL of peripheral blood samples with commercially available kits (NucleoSpin Blood L, Macherey-Nagel, Duren, Germany) according to the manufacturer’s protocol. The extracted DNA was stored at −80 °C until further use. The concentration of the resulting DNA was measured spectrophotometrically at 260 nm by a NanoVue TM Spectrophotometer (Healthcare, Buckinghamshire, UK). The ratio of absorptions at 260 nm vs. 280 nm was calculated to assess the purity of DNA samples.

### 2.3. Genotyping for the TGFB1 -509 C/T Polymorphism (rs1800469)

Genotyping was performed by the polymerase chain reaction-restriction fragment length polymorphism (PCR-RFLP) assay as previously described [24]. Amplification was performed with the 5′–CAGTAAATGTATGGGGTCGCAG–3′ forward primer and 5′–GGTGTCAGTGGGAGGAGGG–3′ reverse primer set at the following cycling parameters: initial denaturation step of 3 min at 94 °C; 30 cycles of 1 min at 94 °C, 1 min at 63.3 °C, and 1 min at 72 °C. Final elongation was performed for 7 min at 72 °C. PCR reactions were carried out on a QCycler (QuantaBiotech Ltd., Kingston upon Thames, UK). The PCR reagents were purchased by Thermo Fisher Scientific (Waltham, MA, USA).

Amplification was visualized on 2% agarose gel electrophoresis. The amplified products (15 μL) were digested with 10U *Eco*81I restriction enzyme (Thermo Fisher Scientific) per reaction at 37 °C overnight. The restriction products were separated on a 3% agarose gel stained with ethidium bromide (0.5 mg/mL). Each PCR run included a heterozygous control template and a negative template control to ensure accuracy.

### 2.4. Quantification of Serum TGF-β1 Levels

Serum concentrations of acid-activated TGF-β1 protein were determined by the quantitative sandwich enzyme-linked immunosorbent assay (ELISA) method according to the manufacturer’s instructions (ElabScience, Huston, TX, USA). The commercially available ELISA kit was provided with a detection range of 0.16–10 ng/mL and a sensitivity of 0.10 ng/mL. Latent serum TGF-β1 was activated with 1 N HCl for 15 min at room temperature, followed by neutralization with 1.2 N NaOH. The activated samples from the patients and controls were analyzed together in the same analytic batch. The results were calculated by reference to the standard curve and expressed as nanograms per ml (ng/mL). The standard curve was constructed using standards provided by the manufacturer within the range of 0 pg/mL to 2000 pg/mL. Results were read on an EZ Microplate Reader (Biochrom, Cambridge, UK).

### 2.5. Immunohistochemistry

Tissue specimens from 39 patients were analyzed immunohistochemically. After fixation in 10% buffered formalin, biopsies were embedded in paraffin and then cut into sections of 4 μm thickness. Next, they were dewaxed, and endogenous peroxidase was blocked for 5 min with a blocking reagent. Slides were washed 3 times with PBS and incubated with primary antibodies for 1 h. After washing 3 times, the slides were incubated with the marked polymer and then washed again. In the last phase, they were incubated with DAB substrate-chromogen and washed again. At the end, they were contrastained with Mayer’s hematoxylin.

Rabbit anti-human TGFβ1 antibody (sc-146, Santa Cruz Biotechnology, Dallas, TX, USA) in a dilution of 1:50 and rabbit anti-human TGFβRII antibody (sc-400, Santa Cruz Biotechnology, USA) in a dilution of 1:50, as well as the detection system EnVision™ FLEX+, Mouse, High pH (Link) (K8002, DAKO, Glostrup, Denmark), were used for the immune reaction. The analysis was performed according to the manufacturer’s protocols, and the final score for TGF-β1 and TGFβRII expression was obtained according to immunostaining intensity in tumor epithelial cells and was designated as negative—score 0, or positive 1+.

### 2.6. Statistical Analyses

Statistics was carried out with SPSS Software, v.25 (IBM, Chicago, IL, USA). The genotype and allele frequencies among the patients and controls, as well as the test for deviation from the Hardy–Weinberg equilibrium, were analyzed by the χ^2^ test. The StatPages.net website (http://statpages.org/index.html, accessed on 30 December 2023) was used to estimate the crude odds ratios (ORs) with 95% confidence intervals (95% CI) for disease susceptibility with respect to the *TGFB1* -509C/T polymorphism. The adjusted odds ratios with a 95% confidence interval were calculated by binary logistic regression, with age and sex as covariates. The Shapiro–Wilk W test was used for analyzing the normality of the distribution. The continuous variables were compared between independent groups by the Student’s *t*-test, or ANOVA. Survival analysis was performed by the Kaplan–Meier method, and a log-rank test was applied for comparisons. All factors with a level of significance of *p* < 0.05 were considered statistically significant.

## 3. Results

### 3.1. Distribution of TGFB1-509C/T Polymorphism

**Figure 1:**
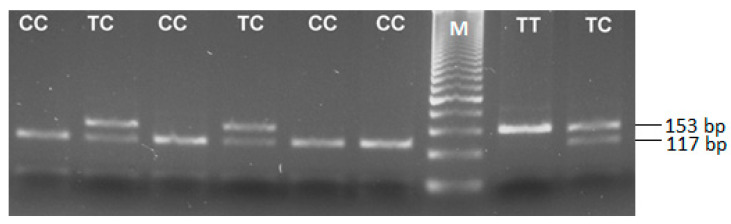
RFLP-PCR agarose gel representing the observed *TGFB1*-509C/T genotypes. The T/T—carriers are visualized with a single band of 153 bp; the C/T—with two bands of 153 bp and 117 bp; and the C/C—with a band of 115 bp (the 38 bp fragment was too short to be detected); M—50 bp DNA ladder.

The specific primers amplified a 153 bp region from the promoter of the *TGFB1* gene. In the presence of the C-allele, an *Eco*81I restriction site is available, generating two fragments of 115 bp and 38 bp. The PCR product of the T-allele is not restricted by the enzyme and is visualized on an agarose gel as a single band of 153 bp. The observed genotypes are illustrated in Figure 1. Individuals homozygous for the C-allele (C/C genotype) were detected with a band of 115 bp, and the 38 bp fragment was too short to be detected. The heterozygous C/T genotype was visualized with two bands of 153 bp and 115 bp, and the T/T genotype with the amplicon’s band of 153 bp.

**Figure 2:**
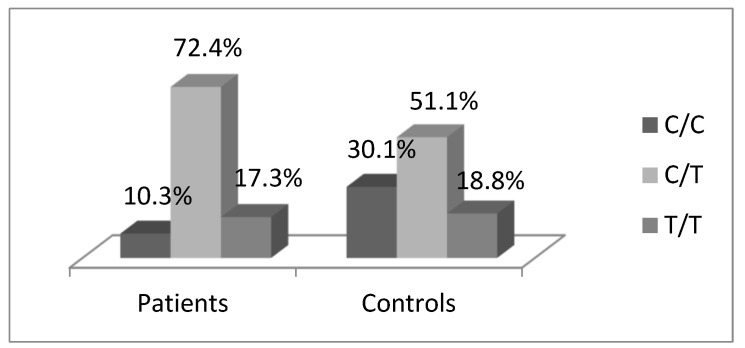
Case-control difference in genotype distributions related to the *TGFB1*-509C/T promoter polymorphism (*p* = 0.005).

A total of 61 patients and 176 healthy individuals were successfully genotyped for the *TGFB1*-509C/T polymorphism. The genotype distributions in the control group were in agreement with the expected values fitting in the Hardy–Weinberg equilibrium (χ^2^ = 3.18, *p =* 0.204). A statistically significant case-control difference in genotype distributions was observed (*p =* 0.005, χ^2^-test). Among the patients, 7 (10.3%) were homozygous for the C-allele, 41 (72.4%) were heterozygous, and 13 (17.3%) were carriers of the T/T genotype. The genotype distribution among the controls was as follows: 53 (30.1%) were C/C carriers, 90 (51.1%)—C/T, and 33 (18.8%)—T/T. (Figure 2, Table 2). Thus, our results indicated that the C-allele was rarer and the heterozygous genotype was overrepresented among the patients.

**Figure 3:**
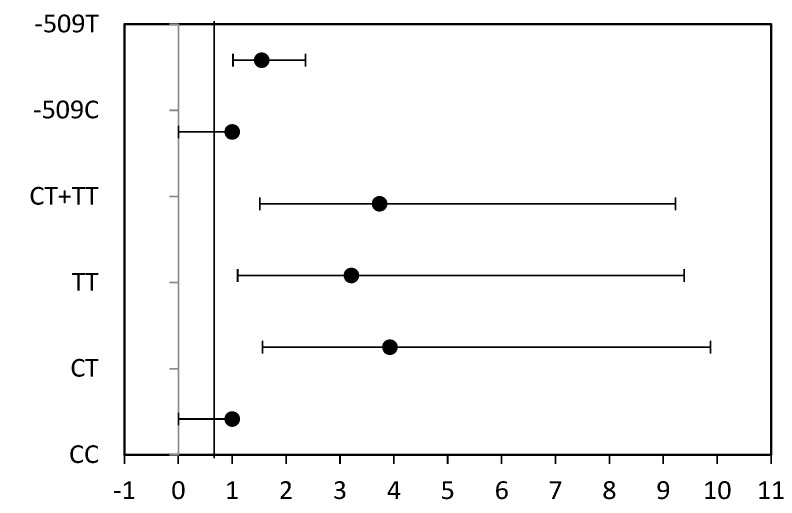
Odds ratio plot represented with 95% CI according to the studied -509C/T SNP.

As shown in Table 2 and Figure 3, a significantly increased risk for brain malignancies was found for CT versus CC (OR = 3.926; 95% CI 1.560 ÷ 9.878; *p* = 0.002), TT versus CC (OR = 3.212; 95% CI 1.100 ÷ 9.384; *p* = 0.037), and CT + TT versus CC (OR = 3.734; 95% CI 1.512 ÷ 9.225; *p* = 0.003). Overall, our results indicate that the -509T allele increased the cancer risk (OR = 1.546; 95% CI 1.014÷2.358; *p* = 0.053).

### 3.2. Survival of the Patients According to the TGFB1-509C/T SNP

Data on the survival status of the patients were available for 48 cases (24 high-grade glial tumors and 24 cases with brain metastases). The follow-up period started from the diagnosis date and included 0 to 45 months. The mean overall survival of glioma patients was 7.99 (±1.84) months and 8.71 (±1.26) for the metastatic cases.

**Figure 4:**
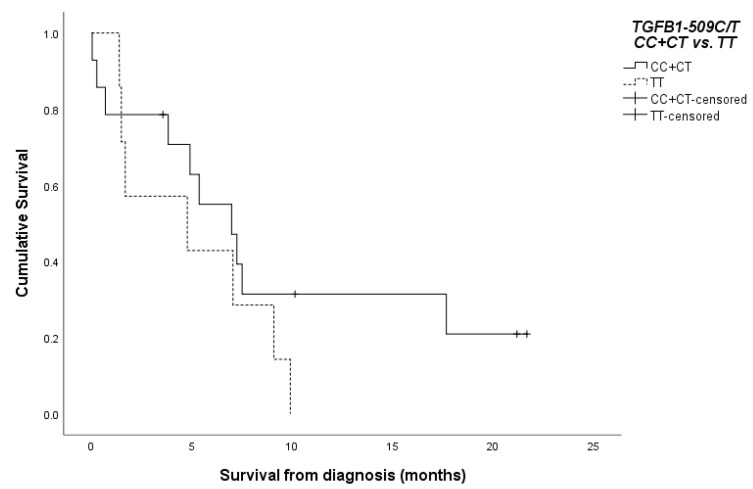
Kaplan–Meier survival plot of the high-grade glioma patients according to *TGFB1*-509C/T genotypes (*p* = 0.257, log-rank test).

The survival rates of the cases with high-grade glial tumors were evaluated with respect to *TGFB1*-509C/T genotypes. It appeared that the carriers of the T/T-genotype had a shorter overall survival of 5.64 (±1.49) months compared to the carriers of the C-allele (C/T or C/C-genotypes)—14.31 (±3.92) months, but without statistical significance (*p* = 0.257, log-rank test). The Kaplan–Meier survival curve is presented in Figure 4.

### 3.3. Serum Levels of TGF-β1

**Figure 5:**
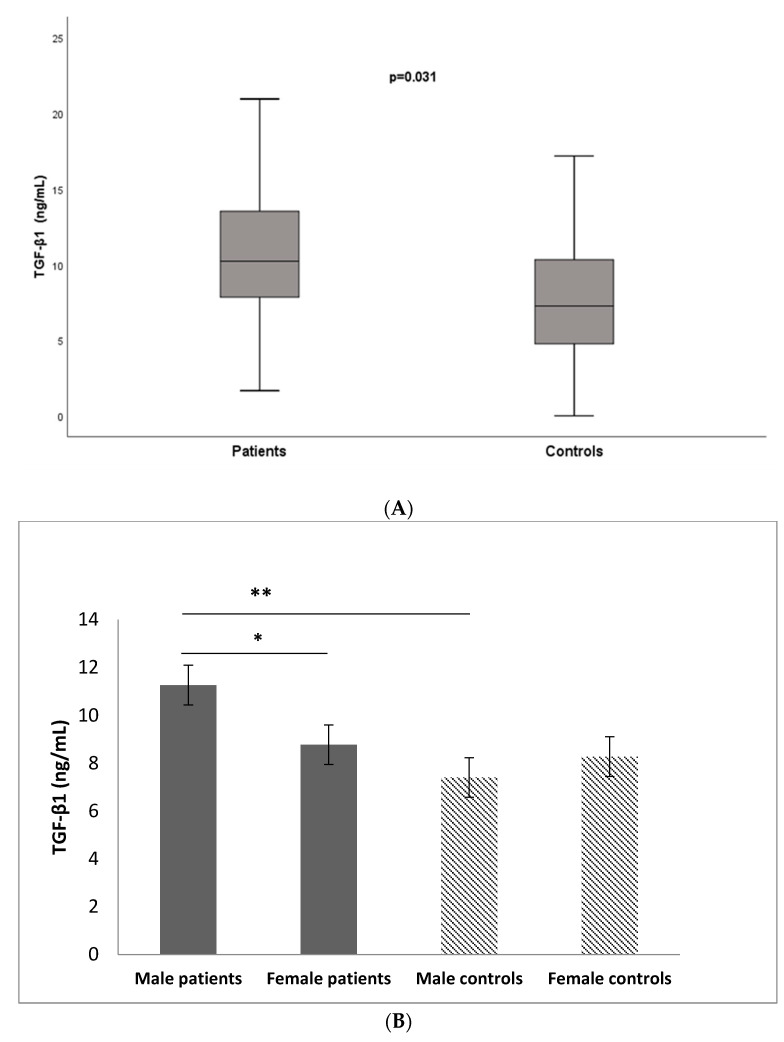
(**A**) Serum levels of TGF-β1 in patients vs. controls (*p* = 0.031, *t*-test); (**B**) Serum levels of TGF-β1 in controls and patients (stratified by sex). * male patients vs. female patients (*p* = 0.055, *t*-test); ** male patients vs. male controls (*p* = 0.025, *t*-test). Data are presented as the mean ± standard error of the mean (SE).

TGF-β1 serum levels were measured in 55 patients with brain tumors (primary glial tumors or metastases) and 31 controls by the ELISA method. Significantly higher serum levels of TGF-β1 in the total patient group (10.28 ± 4.73 ng/mL) were observed compared to the control group (8.06 ± 4.07 ng/mL, *p* = 0.031, *t*-test). We also found a clear tendency that male patients had significantly higher serum levels (11.27 ± 4.43) compared to females (8.78 ± 4.87, *p* = 0.055, *t*-test). Additionally, the male patients had significantly higher TGF-β1 serum levels than the male controls (*p* = 0.009, *t*-test). The data are presented in Figure 5.

**Figure 6:**
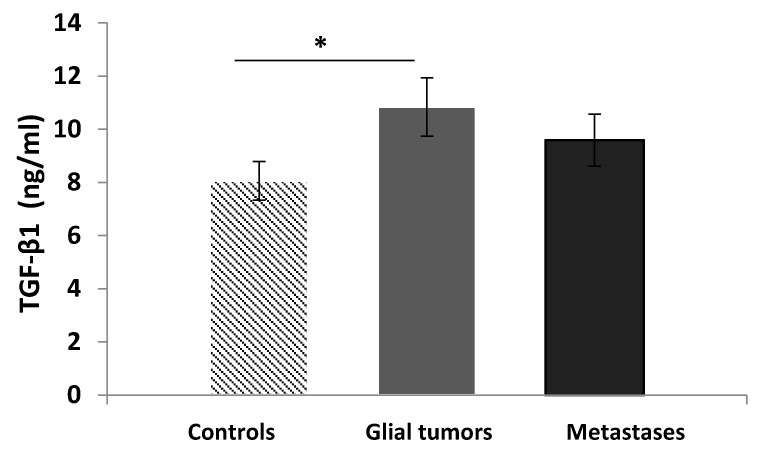
Serum levels of TGF-β1 in controls and patients (stratified by diagnosis). Data are presented as the mean ± standard error of the mean (SE). * controls vs. glial tumors (*p* = 0.033, *t*-test).

After stratification of the patients according to the diagnosis, we found significantly elevated TGF-β1 serum levels in patients with primary glial tumors (10.84 ± 5.06 ng/mL) compared to the control group (8.06 ± 4.07 ng/mL, *p* = 0.033, *t*-test). Among the patients with metastases, the serum levels of the cytokine were slightly elevated (9.59 ± 4.51 ng/mL) compared to the controls but without statistical significance (*p* = 0.222, *t*-test) (Figure 6).

**Figure 7:**
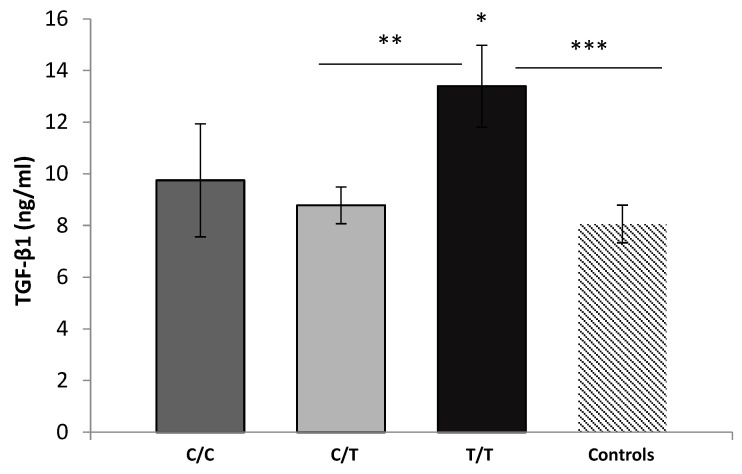
Serum levels of TGF-β1 in controls and patients (stratified by *TGFB1*-509C/T genotypes). Data are presented as the mean ± standard error of the mean (SE). * T/T genotype vs. C/C + C/T (*p* = 0.023, ANOVA); ** T/T vs. C/T (*p* = 0.06, LSD test); *** T/T vs. controls (*p* = 0.011, *t*-test).

According to the studied *TGFB1* rs1800469 SNP, we observed a significant difference in TGF-β1 serum levels in the patients. The homozygous carriers of the ancestral C-allele (C/C-genotype) had a mean TGF-β1 level of 9.75 ± 4.91 ng/mL; the heterozygous carriers (C/T-genotype) had a mean of 8.78 ± 3.93 ng/mL; and the T/T-carriers had the highest TGF-β1 serum levels of 13.39 ± 4.78 ng/mL (*p* = 0.023, ANOVA). The most prominent difference in TGF-β1 serum levels was between the C/T- and T/T-genotypes (*p* = 0.006, LSD test). We also found significantly elevated TGF-β1 concentration in T/T-carriers compared to the controls (*p* = 0.011, *t*-test), Figure 7.

**Figure 8:**
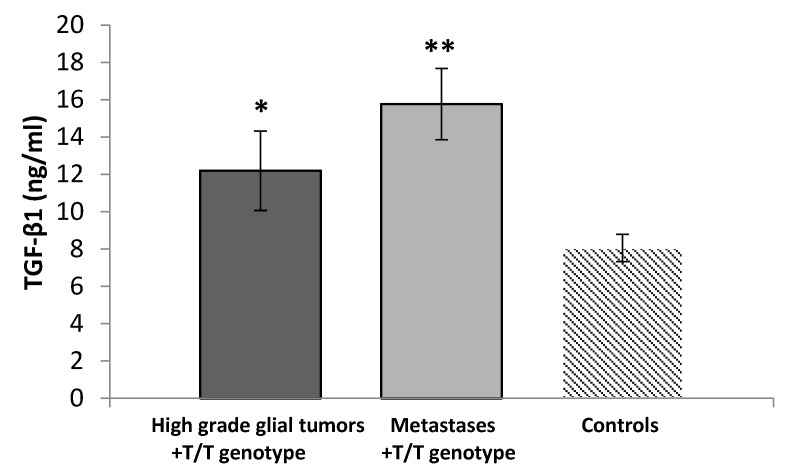
Serum levels of TGF-β1 in controls and patients (stratified by diagnosis + T/T-genotype). Data are presented as the mean ± standard error of the mean (SE). * *p* = 0.036; ** *p* = 0.003.

We also compared the serum concentrations of TGF-β1 in patients with high-grade glial tumors carrying the T/T-genotype with the controls, and there was a significant elevation in the patients (12.20 ± 4.43 ng/mL) vs. the controls (8.06 ± 4.07 ng/mL, *p* = 0.036, *t*-test). A similar result was observed comparing TGF-β1 serum levels in patients with metastases carrying the T/T-genotype (15.77 ± 3.02 ng/mL) to the controls (*p* = 0.003, *t*-test), Figure 8.

### 3.4. Immunohistochemistry

**Figure 9:**
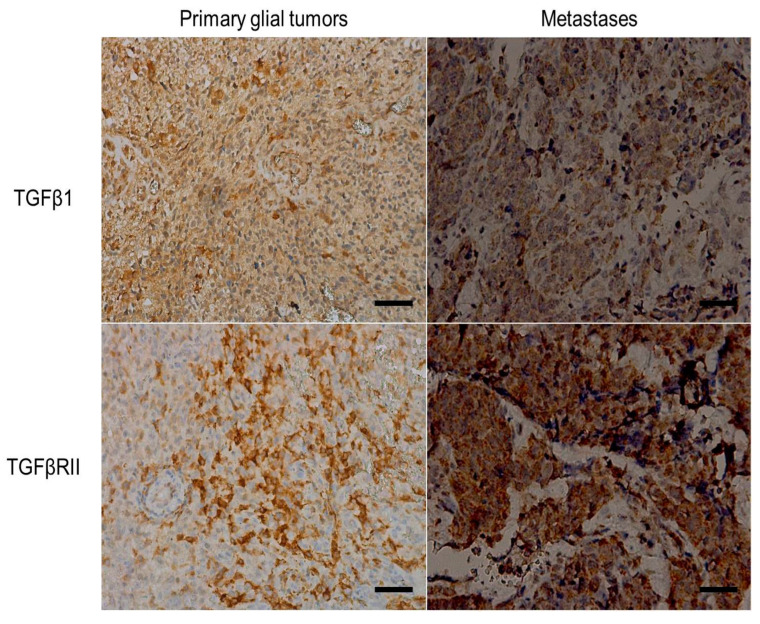
Immunohistochemical expression of TGFβ1 and TGFβRII in tumor tissue of patients with primary glial tumors and metastatic cases; ×200, scale bar = 20 µm.

Immunohistochemistry was performed on 39 tumor specimens (24 cases of primary glial tumors and 15 cases of brain metastases) (Figure 9). The results from the immunohistochemical examination showed that 19 (48.7%) of the patients had cytoplasmic TGF-β1 expression, and 20 (51.3%) were negative. The TGF-β1 receptor type II was expressed on tumor cell membranes in 27 (69.2%) of the cancers. Furthermore, we found that 17 of the tumors expressing TGF-β1 in the cytoplasm also expressed TGFβ-RII (*p* = 0.006, χ^2^-test) (Table 3).

To examine the relationship between cytoplasmic expression and serum levels of TGF-β1, we compared the mean concentrations of the cytokine between the TGF-β1-positive and negative cases. The results showed that cases expressing TGF-β1 in the cytoplasm had elevated levels of the cytokine in the serum (11.97 ± 1.91 ng/mL) compared to the negative cases (8.03 ± 0.66 ng/mL) (*p* = 0.073, *t*-test). The cases expressing TGFβ-RII on the cell membrane also had higher serum levels of the cytokine (10.62 ± 1.2 ng/mL) compared to the negative cases (7.4 ± 1.35 ng/mL) (*p* = 0.094, *t*-test).

## 4. Discussion

Emerging evidence in cancer research demonstrates that the genetic predisposition due to cytokine gene polymorphisms underlies the complex molecular mechanisms of carcinogenesis. Such is the *TGFB1*-509C/T SNP (rs1800469), for which a functional effect on the gene product has been reported. In our study, we found that the heterozygous C/T genotype was more frequent in patients with high-grade glial tumors or brain metastases, determining a higher risk for the patients (OR = 3.926, *p* = 0.002). The combined C/T and T/T genotypes also determined a more than 3-fold increased risk for developing brain cancer conditions (OR = 3.734, *p* = 0.003). Similar results were reported by Wang et al. The authors conducted a case-control study to explore the relationship between three SNPs in the *TGFB1* gene and the development of metastatic brain tumors in non-small cell lung cancer patients [25]. The results demonstrated that individuals carrying the *TGFB1* rs1800469 T/T and C/T + T/T genotypes had an increased risk of developing brain metastasis compared to the C/C genotype, and a significant interaction was observed between the rs1800469 polymorphism and disease stage. Another study on the impact of *TGFB1*-509C/T and 869T/C polymorphisms on glioma risk did not find a significant association between the studied SNPs and glioma risk [26]. Additionally, the latter publication reported that the homozygous -509T/T genotype was associated with longer overall survival of glioblastoma (GBM) patients when compared to patients carrying CC and CT genotypes (OR = 2.41, *p* = 0.036). Inconsistent with these observations, our results indicated a shorter overall survival after operation for the carriers of the T/T-genotype with high-grade glial tumors compared to the carriers of at least one C-allele, although it should be noted that the survival functions did not find statistical significance. Nevertheless, the negative effect of the T-allele on overall patient survival has been reported widely in other types of cancer, such as colorectal [24], non-small-cell lung cancer [27], and gastric cancer [28].

As the secretion of TGF-β1 is genetically determined, we tested the association between the rs1800469 genotypes and serum levels of the cytokine in the genotyped patients. Our study demonstrated that the T/T-genotype correlates with elevated TGF-β1 circulating levels in patients with brain cancers compared to the carriers of the other two genotypes (*p* = 0.023) and compared to the controls (*p* = 0.011). Moreover, the T/T-carriers with brain metastases had the highest serum concentration (15.77 ng/mL) than the healthy individuals (8.06 ng/mL, *p* = 0.003). We also compared the serum TGF-β1 concentrations of the patients with those in the control group (8.06 ± 4.07 ng/mL), which showed a significant elevation of the serum levels in the patients (10.28 ± 4.73 ng/mL, *p* = 0.033), and the association appeared to be gender-related. Namely, TGF-β1 had the highest concentration in males compared to female patients and healthy individuals. 

The functional role of the *TGFB1*-509C/T SNP in the circulating levels of the cytokine has been previously reported. In a classical twin study, -509T homozygous individuals had almost double the level of TGF-β1 in plasma when compared to -509C homozygous individuals [29]. Recently, the molecular mechanism of the effect of this genetic variation on *TGFB1* gene expression has been elucidated. It was shown that the transcription factor AP1 (c-Fos/JunD) was exclusively recruited to the gene promoter in vitro and in vivo only in -509C carriers [8], suppressing the expression of the gene product. In our study, we demonstrate that the higher-producing genotype is homozygous for the variant T-allele. By determining elevated circulating levels of the cytokine, we suggest that the T/T-genotype is a predisposition for developing brain cancer and probably creates a metastatic niche in the transforming cells, as previously reported [30]. Our results correspond to other studies on the association of elevated serum levels of TGF-β1 with the clinical prognosis for pancreatic cancer [31], gastric cancer [32], and osteosarcoma [33]. Additionally, recent findings indicate that TGF-β1 increases the expression of genes encoding key enzymes of the glycolytic pathway, including PFKFB3, inducing glycolisis in the glioblastoma cell line T98G and in the malignant glioma cell line U87-MG, presenting conclusive evidence on the pro-tumorigenic properties of TGF-β1 in transforming cells. It appears that the upregulation of PFKFB3 by TGF-β1 takes place at a transcriptional level and does not require de novo protein synthesis [34].

TGF-β1 signaling is involved in a myriad of cellular processes and is therefore a key regulator of carcinogenesis. However, its role remains to be fully elucidated, mainly due to the dichotomous effects of TGF-β1 in different cancers: although it can suppress tumorigenesis in the early stages, it promotes tumor growth in the late stages of the disease [35,36]. This balance is likely regulated by the presence of the TGFβ receptors—types I, II, and III. Data regarding TGFβ-RII tissue expression and its role in tumorigenesis are relatively scarce. As a significant part of the TGFβ signaling pathway, some authors describe that alterations in TGFβ-RII levels lead to a change in the inflammatory response as well as the possibility of the development and modulation of tumor processes [37]. Such literature data are available for epithelial gastrointestinal tumors as well as some endocrine tumors [38,39]. In our study, we show that cases expressing both the receptor and the TGF-β1 molecule have significantly higher serum levels of the cytokine. Therefore, we suggest that the availability of the receptor in the membrane enhances the recruitment of TGF-β1, leading to an altered immune response that might promote tumor growth and metastasis.

## 5. Conclusions

Conclusively, the above-discussed data suggest that the *TGFB1*-509C/T SNP might be a genetic factor responsible for the predisposition and prognosis of primary brain tumors and brain metastases. Further analysis of the molecular bridges that bound circulating TGF-β1 levels and other factors in the complex network of the tumor microenvironment favoring tumor growth and metastases is required. Although the major limitation of the present study is the small sample size, our results add novel insights into the prognostic relevance of the studied polymorphism to the tumorigenicity of TGF-β1.

## Figures and Tables

**Table 1:** Main clinical and histological parameters of the studied patients.

Parameter	Number (%)
Patients	n = 61
Gender	
Male	40 (65.6)
Female	21 (34.4)
Age at diagnosis (years)	
Range	25–82
Mean (±SD)	62.1 (±11.76)
<62.1	27 (44.3)
>62.1	34 (55.7)
Primary/Metastases	
Primary glial tumors	31(50.8)
Grade III	2 (3.2)
Grade IV	29 (47.6)
Metastases	30 (49.2)
Chemotherapy	
yes	52 (85.2)
no	9 (14.8)
Radiotherapy	
yes	28 (45.9)
no	33 (54.1)
Survival status	n = 48
alive	15 (31.2)
deceased	33 (68.8)
TGF-beta1 expression	n = 39
positive	19 (48.7)
negative	20 (51.3)
TGF-beta RII expression	n = 39
positive	27 (69.2)
negative	12 (30.8)

**Table 2:** The genotype and allelic frequencies of the -509C/T SNP in the *TGFB1* gene promoter among patients and healthy individuals.

*TGFB1*-509 C/T SNP	Patients		Controls		OR (95% CI), *p*-Value	OR * (95% CI), *p*-Value
	n	Frequency	n	Frequency		
	61		176			
Genotype						
CC	7	0.10	53	0.30	1.0 (Ref.)	1.0 (Ref.)
CT	41	0.73	90	0.51	3.926 (1.560–9.878), *p =* 0.002	4.292 (1.092–9.685), *p =* 0.002
TT	13	0.17	33	0.19	3.212 (1.100–9.384), *p =* 0.037	3.215 (1.060–9.110), *p =* 0.033
CT + TT	54	0.90	123	0.70	3.734 (1.512–9.225), *p =* 0.003	3.968 (1.010–9.624), *p =* 0.003
Alleles						
-509C	35	0.47	196	0.56	1.0 (Ref.)	…
-509T	46	0.53	156	0.44	1.546 (1.014–2.358), *p =* 0.053	…

OR, odds ratio; * adjusted for sex and age; CI, confidence interval; …, not applicable.

**Table 3:** Immunohistochemical expression of TGF- β1 and TGFβ-RII in patients.

	TGF-β1 Expression	TGFβ-RII Expression	TGF-β1/TGFβ-RII Co-Expression	*p*
Positive	19	27	17	0.006
Negative	20	12	-	-

## Data Availability

Data are contained within the article.
